# Multiplexing LAMP Assays: A Methodological Review and Diagnostic Application

**DOI:** 10.3390/ijms25126374

**Published:** 2024-06-09

**Authors:** Beatriz Crego-Vicente, Manuel Diego del Olmo, Antonio Muro, Pedro Fernández-Soto

**Affiliations:** Infectious and Tropical Diseases Research Group (e-INTRO), Biomedical Research Institute of Salamanca Research Centre for Tropical Diseases at the University of Salamanca (IBSAL-CIETUS), Faculty of Pharmacy, University of Salamanca, 37007 Salamanca, Spain; beatrizcregovic@usal.es (B.C.-V.); manueldiego@usal.es (M.D.d.O.)

**Keywords:** LAMP, primers, multiplex LAMP, DARQ-LAMP, QUASR-LAMP, dry-LAMP, fluorophore, probe, diagnostic, point-of-care

## Abstract

The loop-mediated isothermal amplification (LAMP) technique is a great alternative to PCR-based methods, as it is fast, easy to use and works with high sensitivity and specificity without the need for expensive instruments. However, one of the limitations of LAMP is difficulty in achieving the simultaneous detection of several targets in a single tube, as the methodologies that allow this rely on fluorogenic probes containing specific target sequences, complicating their adaptation and the optimization of assays. Here, we summarize different methods for the development of multiplex LAMP assays based on sequence-specific detection, illustrated with a schematic representation of the technique, and evaluate their practical application based on the real-time detection and quantification of results, the possibility to visualize the results at a glance, the prior stabilization of reaction components, promoting the point-of-care use, the maximum number of specific targets amplified, and the validation of the technique in clinical samples. The various LAMP multiplexing methodologies differ in their operating conditions and mechanism. Each methodology has its advantages and disadvantages, and the choice among them will depend on specific application interests.

## 1. Introduction

Molecular diagnostics, especially qPCR, allow for the simultaneous detection and differentiation of microbial pathogens. They offer clinical laboratories a rapid, sensitive and specific alternative to many phenotype-based biochemical reactions, significantly reducing the time required for analysis per sample [1]. Numerous PCR-based diagnostic tests are currently available for a wide range of pathogens, including bacteria, fungi, viruses and parasites [2]. Notwithstanding its well-known advantages, PCR also has some limitations. The occurrence of false negatives is possible due to the presence of inhibitors in human specimens that compromise DNA amplification. False positive results can also occur due to contamination or mismatched target amplifications. Thus, in order to reduce or eliminate potential false negative or positive results, extreme care should be taken in PCR setup and optimization, as well as in result interpretation [3]. Additionally, PCR requires expensive thermocyclers that provide precise and extreme temperature cycles demanding uninterrupted power sources as well as trained personnel. Along with the mandatory reagents, consumables, and storage facility, PCR-based assays are largely restricted to well-resourced or centralized clinical laboratories [4], presenting a technical challenge for developing point-of-care (POC) diagnostics.

**Figure 1:**
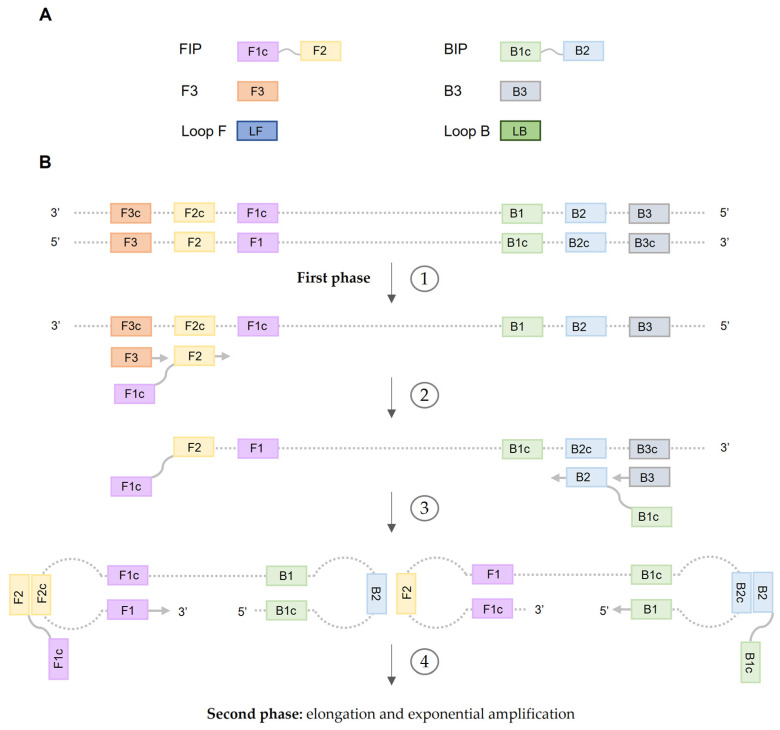
Loop-mediated isothermal amplification (LAMP): primers and mechanism. (**A**) A set of LAMP primers in the LAMP reaction is represented. LAMP reaction requires four primers, two inner primers (forward inner primer (FIP) and backward inner primer (BIP)) and two outer primers (F3 and B3). FIP and BIP each contain two sequences (usually linked by a poly-T linker) corresponding to the sense and antisense sequences of the target DNA. Additional loop primers (loop-forward (LF) and loop-backward (LB)) can be included, shortening the reaction time up by approximately 30 min. (**B**) LAMP amplification occurs in two phases. In the first phase: 1. FIP (F1c-F2) hybridizes to the partially denatured template DNA and is elongated by the *Bst* polymerase. 2. The outer primer F3 anneals to the same single-stranded DNA (ssDNA) template strand and is elongated by *Bst* polymerase. Now, the ssDNA FIP-elongation product is displaced. That ssDNA is used as template for the backward primers. The inner primer BIP (B1c-B2) hybridizes and starts strand synthesis at the ssDNA and then is displaced by the B3 primer. 3. As the 3′ and 5′ ends are complementary to sequences further inwards, terminal stem-loop DNA structures are formed and subsequently used as targets to start an exponential amplification second phase. 4. In the second phase, self-priming and the elongation of 3′ end induces the displacement of the 5′ end and, subsequently, the hairpin comes off and the newly synthesized strands are folded. Further self-priming repetitions generate many amplicons with cauliflower-like structures. In addition, FIP and BIP primers now hybridize to the loop structures formed and initialize strand synthesis and subsequent displacement.

Isothermal nucleic acid amplification techniques (iNAATs) were developed in the early 1990s with the goal to overcome the limitations of PCR technologies. Since isothermal amplification can be performed at one reaction temperature under simple conditions (e.g., in a water bath or heating block) it facilitates rapid and accurate molecular diagnosis at very reduced cost [5,6]. Among the iNAATs established, the loop-mediated isothermal amplification (LAMP) assay has become one of the most popular methods among researchers [7]. LAMP was first described by Notomi et al. in 2000 [8] and was patented by Eiken Chemical Co., Ltd. (http://www.eiken.com.cn/) (accessed on 7 February 2024). LAMP is based on auto-cycling strand displacement DNA/RNA synthesis performed under isothermal conditions (60–65 °C for 45–60 min) using a *Bst* polymerase with strand displacement activity [8]. The LAMP reaction requires 4 to 6 primers, capable of recognizing 6 to 8 specific regions of the target DNA/RNA sequence, thus conferring high specificity for amplification. The principle of LAMP is depicted in Figure 1. In addition, there are a large number of possibilities and systems for reading and analyzing the results of LAMP amplifications, including end-point analysis by agarose gel electrophoresis [8], simple naked eye colorimetric visualization by intercalating dyes [9] or metal indicators [10], as well as real-time detection in a number of devices by turbidity [11], color change in fluorescence metal-sensitive indicators [9], unselective DNA fluorescent intercalating dyes [12] or target specific-fluorogenic probes [7]. Several approaches, such as lateral flow assays (LFA) [13] and novel combinations with CRISPR-Cas Systems [14] have also been developed. Moreover, the ability to stabilize reaction mixes through drying protocols (dry-LAMP) or lyophilization, enables all necessary reagents to be kept at room temperature until use, thus eliminating the need for cold chain maintenance. This contributes to reducing the required equipment in the field, allowing molecular diagnostics to be brought closer to low-resource areas as POC [15,16,17]. However, LAMP presents a number of limitations that need to be acknowledged, such as non-applicability for cloning, the primer design being subject to more constraints than other iNAAT, a high risk of carry-over contamination, and the multiplexing approaches for multiple pathogen detection are highly complex and still underdeveloped [18].

Real-time fluorescence is currently a widely used method for LAMP result detection, offering high sensitivity, compatibility with most standard isothermal amplification devices available in labs, and usefulness for optimizing and verifying the kinetics of amplification reactions. Specific sequence detection using fluorogenic probes ensures exceptional specificity towards the target, avoiding the detection of the non-specific products responsible for false positives and also enabling the simultaneous detection of multiple targets (multiplexing) in a LAMP assay [7]. Multiplex LAMP (mLAMP) has great potential for the rapid and accurate diagnosis of infectious diseases, enabling not only the differentiation of multiple pathogen species but also closely related strains or species in a single assay [9,19]. This is of great interest, as approximately 30% of human infections may actually be coinfections, and this rate could be as high as 80% in some human communities [20,21]. Various methods for multiplexing LAMP use fluorophore-labeled probes attached to complementary oligonucleotide sequences [7]. However, the type of fluorophore used and interactions between fluorophores and primers can negatively impact fluorescence emission, requiring the adaptation of fluorophore-labeled probes to different target sequences. This may be a weakness of the methodology, as it involves significant time for primer optimization and probe design to fine-tune the mLAMP assay. Consequently, the limited and poorly reproducible studies on fluorescence-based multiplexed LAMP detection may be due to these challenges. Due to the increased complexity of in silico design compared to simplex LAMP, there is no standardized methodology for the development of a multiplex LAMP assay [22]. To date, a wide variety of different methods have been used, including those that utilize modified primers, universal probes, restriction enzymes, nanoparticles, the combined use of LAMP with other techniques, melting curve analysis, agarose gel electrophoresis, and microfluidic chip technologies.

In this review, we present these methods for the development of multiplex LAMP assays, illustrated with a schematic representation of the technique, and assess their practical application based on the following characteristics: (i) real-time detection and quantification of results; (ii) the possibility of visualizing results at a glance, allowing for easy and rapid interpretation; (iii) prior stabilization of reaction components, enabling the elimination of the cold chain and promoting point-of-care (POC) use; (iv) maximum number of specific targets amplified; and (v) validation of the technique in clinical samples. It should be noted that, to date, none of the developed multiplex LAMP assays have been evaluated in field studies in low-resource endemic areas.

## 2. Multiplexing LAMP Assays

### 2.1. Methods Using Modified Primers

These methods use specific LAMP assay primers, which incorporate certain modifications to distinguish the specific targets intended for amplification. Typically, these modifications involve the incorporation of different fluorophores (with specific excitation and emission wavelengths) and quenchers (signal attenuators) that deactivate the generated fluorescence signals [23]. Thus, using different fluorophores for each specific LAMP assay enables the differentiation of various targets. Some of the methodologies that utilize modified primers include DARQ (Detection of Amplification by Release of Quenching), QUASR (Quenching of Unincorporated Amplification Signal Reporters), FLOS (Fluorescence of Loop primer upon Self-dequenching), Guanine quenching, and Assimilation Probes.

#### 2.1.1. DARQ 

**Figure 2:**
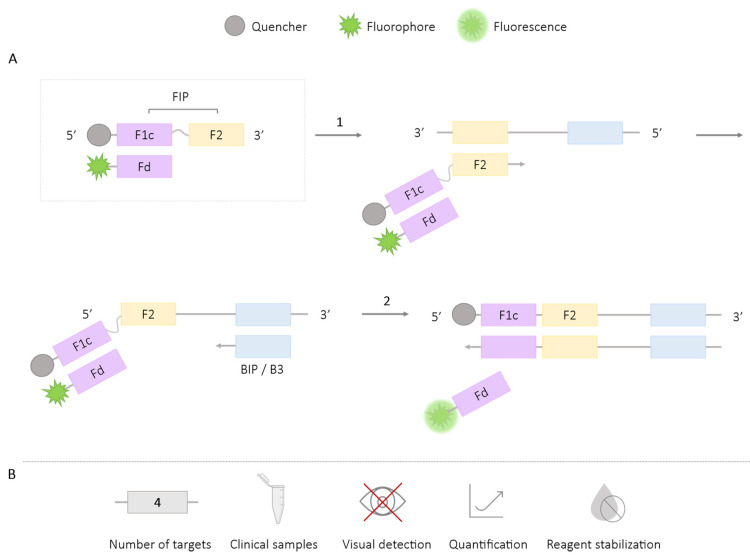
DARQ-LAMP Methodology. (**A**) Schematic representation of the DARQ methodology. The box depicts the FIP primer labeled with the quencher at the 5′ end and the complementary probe to F1c (Fd) labeled with the fluorophore (QPD; Quencher Probe Duplex). 1. Hybridization of labeled primer and Fd, followed by LAMP reaction. 2. Release of Fd-fluorophore probe and fluorescence emission. (**B**) Application (maximum number of amplified targets and use of clinical samples), amplification detection (visual at the end of the reaction or real-time quantitative), and adaptation to a ‘ready-to-use’ format (reagent stabilization).

In the DARQ-LAMP technique, one of the internal primers (for example, FIP) is modified at its 5′ end with a quencher (Q), and a complementary sequence (Fd) is designed to a region of the labeled primer (F1c), which is modified with a fluorophore (F) at its 3′ end. The hybridization of the labeled primer and the complementary sequence generates a duplex structure or probe (QPD, Quencher Probe Duplex), which continues to function as a primer. As the amplification occurs during the course of the reaction, the sequence with the fluorophore is released, generating a detection signal for amplification by separating the fluorophore from the quencher [24,25,26,27,28] (Figure 2). The generated fluorescence signal is detected by real-time fluorescence reading devices, allowing for the quantification of the result.

The DARQ-LAMP methodology has enabled the detection of up to four targets for the simultaneous amplification of *Escherichia coli*, *Caenorhabditis elegans*, bacteriophage λ, and the hBRCA1 gene using genomic DNA from different organisms [24]. It has also been used for the simultaneous detection of *Plasmodium* spp., *P. vivax*, *P. falciparum*, and the human actin gene in blood samples from patients [29]. Furthermore, it has been applied for the simultaneous detection of three methicillin-resistant *Staphylococcus aureus* genes (fem B, mecA, and spa) using genomic DNA samples [26], and for the unique detection of *Salmonella* spp. in stool samples [27]. Recently, it has been also applied for the simultaneous detection of two helminth parasites: *Schistosoma mansoni* and *Strongyloides* spp. [28]

#### 2.1.2. QUASR 

**Figure 3:**
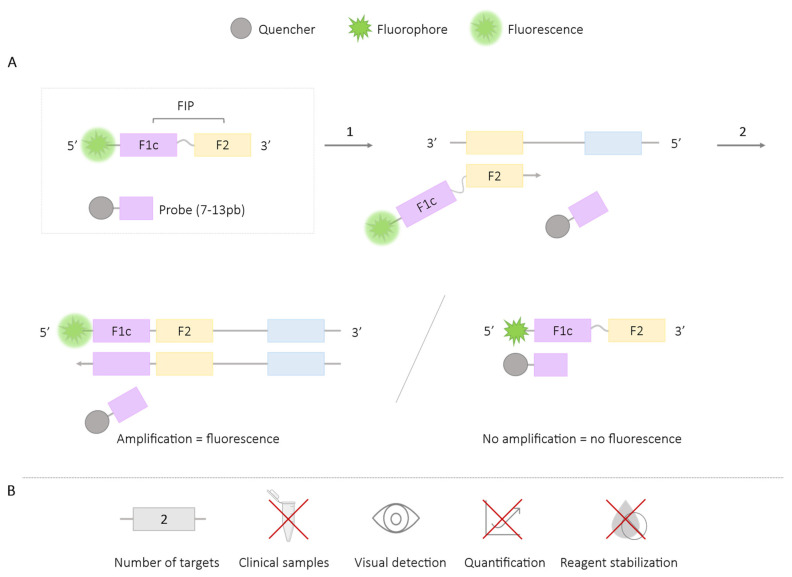
QUASR-LAMP Methodology. (**A**) Schematic representation of the QUASR methodology. The box depicts the FIP primer labeled with the fluorophore at the 5′ end and the complementary probe (7–13 bp) to F1c labeled with the quencher. 1. Hybridization of the FIP primer and LAMP reaction. 2. Reduction in the reaction temperature to room temperature: if amplification occurs, a fluorescence signal is generated; if there is no amplification, no fluorescence signal is generated. (**B**) Application (maximum number of amplified targets and use of clinical samples), amplification detection (visual at the end of the reaction or real-time quantitative), and adaptation to a ‘ready-to-use’ format (reagent stabilization).

In the QUASR-LAMP method, an internal primer or a loop primer is modified at its 5′ end with a fluorophore, and a complementary probe to F1c (containing 7–13 base pairs; bp) is designed, which is modified at its 3′ end with a quencher. Both structures are separately introduced into the mixture, and their hybridization temperature should be lower than that of the LAMP reaction, ensuring they remain dissociated during amplification. After the amplification reaction, the mixture is cooled to room temperature, and if there is no amplification (a negative result), the primer will hybridize with the probe. If there is amplification (a positive result), the primer will be bound to the amplicon, rendering it inaccessible to the probe-quencher structure and thus generating a fluorescence signal [30,31] (Figure 3).

The QUASR-LAMP methodology is limited to an endpoint signal after amplification, not allowing for real-time detection. The results are visualized colorimetrically by combining the excitation of the reaction mixture with a specific wavelength LED light and the use of different photographic filters [30,31]. Smartphone applications have been developed to enhance color discrimination for aiding in objective result interpretation [31,32].

The use of this technique has enabled the simultaneous detection of two viruses in the same reaction: West Nile Virus and Chikungunya [30], Yellow Fever Virus and Dengue [33], and Zika and Chikungunya [31]. Extending its application to a greater number of targets could pose challenges in differentiation due to the mixing of colors generated by overlapping fluorescence signals. The QUASR methodology has not yet been applied to clinical samples.

#### 2.1.3. FLOS 

**Figure 4:**
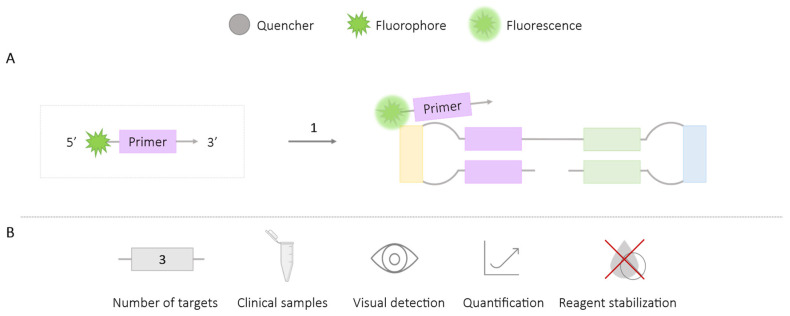
FLOS-LAMP Methodology. (**A**) Schematic representation of the FLOS methodology. The box depicts the primer labeled with the self-quenching fluorophore. 1. LAMP reaction; if amplification occurs, self-activation of the fluorophore produces fluorescence. (**B**) Application (maximum number of amplified targets and use of clinical samples), amplification detection (visual at the end of the reaction or real-time quantitative), and adaptation to a ‘ready-to-use’ format (reagent stabilization).

The FLOS-LAMP methodology is based on self-regulation of the fluorescence signal. Initially, an internal or loop primer is modified at its 5′ end with a fluorophore, which, when freely present in the reaction medium, remains ‘self-quenched’. During the amplification process, the modified primer becomes incorporated into the generated amplicon, causing the fluorophore to dissociate and emit fluorescence (Figure 4). The mechanism of quenching and autofluorescence is not yet well understood, but a possible explanation is based on interactions with the nucleobases present in the reaction medium [34].

Fluorescence emission is generated as the reaction progresses, allowing real-time monitoring. The ability to use different fluorophores has enabled the differentiation of three species of whitefly, *Trialeurodes vaporariorum*, *Bemisia tabaci* (MEAM1), and *B. tabaci* (MED), in a single reaction [35], and the simultaneous detection of two fungi, *Fomitiporia torreyae* and *Fulviformes umbrinellus* [36]. Despite not inherently facilitating colorimetric result detection, a post-reaction addition of polyethyleneimine (PEI) has been used to precipitate the amplified products after brief centrifugation, resulting in a visually observable pellet under UV light emission [35,36]. To date, this methodology has been employed in various clinical samples (bronchoalveolar lavage fluid, urine, saliva, nasal wash, naso/oropharyngeal swabs) for the detection of varicella-zoster virus [34].

#### 2.1.4. Guanine Quenching

**Figure 5:**
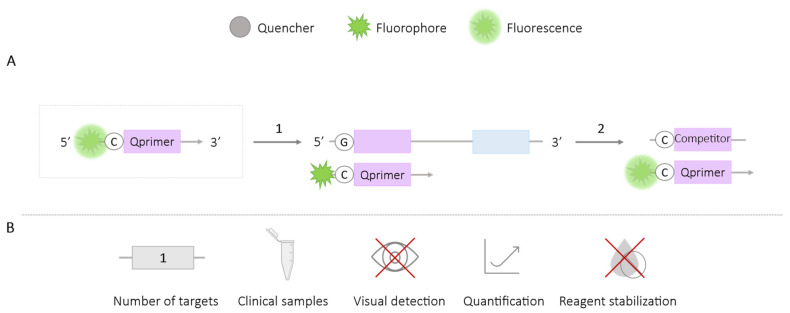
Guanine Quenching-LAMP Methodology. (**A**) Schematic representation of the Guanine Quenching methodology. The box depicts the QPrimer: a primer with a cytosine residue at the 5′ end labeled with a fluorophore. 1. LAMP reaction; if amplification occurs, the fluorescent signal is quenched when the primer binds to the target region with a guanine residue. 2. An alternative using a complementary probe to the QPrimer (competitor) that maintains the fluorescence signal by having a cytosine residue. (**B**) Application (maximum number of amplified targets and use of clinical samples), amplification detection (visual at the end of the reaction or real-time quantitative), and adaptation to a ‘ready-to-use’ format (reagent stabilization).

In the guanine quenching method, the chosen primers for labeling must have a cytosine at the 5′ end, where an adjacent fluorophore (QPrimer) is placed. When the QPrimer hybridizes with the target nucleotide sequence, fluorescence is quenched due to the electron transfer between the fluorophore and the guanine residue present in the target sequence (Figure 5A). This decrease in fluorescence signal can be monitored in real time. The requirement for primers with cytosine residues at the ends poses a limitation for the application of this methodology [37,38].

This methodology can be complemented with the use of a competitive sequence that allows for the quantification of the amount of DNA. Thus, the reaction mixture will contain two targets to which the QPrimer can bind: the target region to be amplified and a complementary probe to the QPrimer, called the competitor (Figure 5). The competitor will have a cytosine instead of a guanine residue, so when it binds to the QPrimer, the signal is not quenched, allowing for the calculation of the amount of product based on the generated fluorescence intensity [39].

This methodology has been employed for the detection of a single amplification target for *Nitrosomonas europaea* in bacterial culture samples [39], Influenza virus (IV), Respiratory Syncytial Virus (RSV) [37], and Middle East Respiratory Syndrome Coronavirus (MERS-CoV) [40]. For IV and RSV [37] and MERS-CoV [40], the technique has been validated in clinical samples from patients (aspirates, nasal secretions, and swabs collected from nasal samples of patients). The possibility of using different fluorophores in primer labeling suggests its potential future development in a multiplex format.

#### 2.1.5. Assimilation Probes or Primer Fluorescence Probes

**Figure 6:**
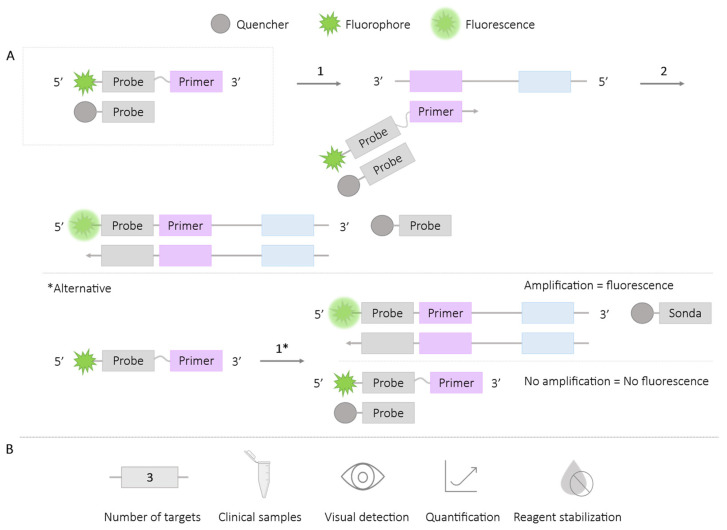
Assimilation-LAMP Probe Methodology. (**A**) Schematic representation of the functioning of the Assimilation-LAMP probe methodology. In the box, the primer is labeled with a probe and a fluorophore at the 5′ end, and a complementary probe (to the primer-bound probe) with a quencher at the 3′ end; both structures are attached at the beginning of the reaction. 1. Binding of the labeled primer and probe and LAMP reaction. 2. Release of the probe-quencher and fluorescence emission. *Alternative: Only the primer labeled with a probe and a 5′ fluorophore are incorporated at the beginning of the reaction. 1*. LAMP reaction and post-reaction addition of the probe-quencher. If there is amplification, a fluorescence signal is generated; if there is no amplification, no fluorescence signal is generated. (**B**) Application (maximum number of amplified targets and use of clinical samples), detection of amplification (visual at the end of the reaction or quantitative in real-time), and adaptation to ‘ready for use’ format (reagent stabilization).

The primer labeling process is similar to the DARQ technology (see Section 2.1.1). In this case, the 5′ end of the primers is labeled with a shorter sequence (probe) with an adjacent fluorophore. Additionally, a complementary probe is designed with a quencher at the 3′ end. This duplex structure remains hybridized and does not generate a signal until amplification occurs. During amplification, based on the strand displacement principle carried out by the *Bst* polymerase, the quencher-containing probe is released, generating a real-time monitored fluorescence signal [41,42,43,44] (Figure 6). The post-reaction fluorescence signal can be observed with the naked eye using LED or UV light, using color photographic filters [43,45,46]. Another possibility is the post-reaction addition of PEI polymer, which precipitates the products, generating a pellet observable under UV light and varying in color depending on the fluorophores used [47].

Following the same principle for primer labeling, another possibility is to add the probe-quencher post-reaction. In the absence of the target, the labeled primer will bind to the probe-quencher, quenching the fluorescence. Conversely, when amplification occurs, the primer will be inaccessible to the probe-quencher, and the fluorescence signal will be maintained (Figure 6A; *Alternative). However, this option does not allow for real-time reading since the result is obtained post-reaction [48,49,50].

This methodology has facilitated the detection of up to three viruses in a single reaction, including Zika, Dengue, and Chikungunya [43,45], validating the technique in clinical urine samples [43]. It has also enabled the simultaneous detection of two targets for *Salmonella enterica* and *Enterobacteria* phage λ in bacterial culture samples [42], *Ralstonia solanacearum* and *R. solanacearum* R3B2 in bacterial culture samples [41,42], SARS-CoV-2 and the human ARNase P gene, as well as validating the technique in nasopharyngeal and saliva samples from patients [46], distinguishing the sex (based on chromosomal regions) of cattle embryos [47], and distinguishing between cow and sheep milk (based on the mitochondrial cytochrome b gene) [44]. Furthermore, several studies have employed this methodology for the detection of HIV (Human Immunodeficiency Virus) [48,49,50] in clinical plasma and blood samples from patients [48,50].

On the other hand, some of the studies using assimilation probes have managed to stabilize the reagents through lyophilization processes, allowing them to forgo the cold chain for storage [43,45,46,49,50]; in some cases, the functionality of the reaction has been maintained for up to one month after reagent storage [49].

### 2.2. Methods Using Universal Probes

These methods utilize universal DNA probes, which are sequences distinct from the specific primers designed for each LAMP assay. Some of them include One-step Strand Displacement (OSD) Probes, Molecular Beacons, and Mediator Displacement (MD) Probes.

#### 2.2.1. One-Step Strand Displacement (OSD) Probes

**Figure 7:**
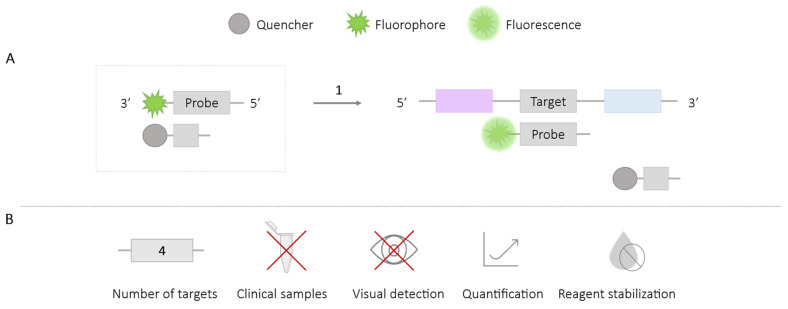
OSD-LAMP Probe Methodology. (**A**) Schematic representation of the OSD probe methodology. In the box, the universal probe labeled with a fluorophore at the 3′ end and the complementary probe labeled with a quencher are depicted. 1. LAMP reaction; binding of the fluorophore-labeled probe to the target if there is amplification and fluorescence emission. (**B**) Application (maximum number of amplified targets and use of clinical samples), detection of amplification (visual at the end of the reaction or real-time quantitative), and adaptation to a ‘ready-to-use’ format (reagent stabilization).

An OSD probe is composed of two complementary sequences, one for binding to the target DNA region labeled with a fluorophore at one end (5′ or 3′), and another complementary to the first one, labeled with a quencher. To promote the binding of the fluorophore-labeled probe to the target region and create the separation of the quencher-labeled probe, the former must have a higher number of base pairs to favor the enthalpy of binding to the target (Figure 7). Therefore, the design of this type of probe must take into account the enthalpy of binding values of the sequences to ensure binding exchange [51,52,53]. The fluorescence signal generated is monitored by real-time fluorescence reader equipment.

The use of the OSD-LAMP probe methodology has enabled the amplification of a single target for the polymorphic detection of a mutant allele in the BRAF oncogene (V600E) [51], the detection of *Wolbachia* in *Aedes aegypti* mosquito specimens [54], the detection of MERS-CoV in cell culture samples [55], and the detection of the Bacteroides HF183 marker as an indicator of human fecal contamination in water samples [52]. It has also allowed for the simultaneous detection in the same reaction of *Plasmodium falciparum* and Herpes Simplex Virus 1 (HSV-1) in artificial samples prepared with genomic material from both organisms [52], and the detection of up to four genetic variants of the Zika virus from infected *Aedes aegypti* mosquito specimens [56].

The use of lyophilization processes for stabilizing reagents has been tested with this methodology, achieving a reagent viability of up to one hundred days after storage [52].

#### 2.2.2. Molecular Beacon

**Figure 8:**
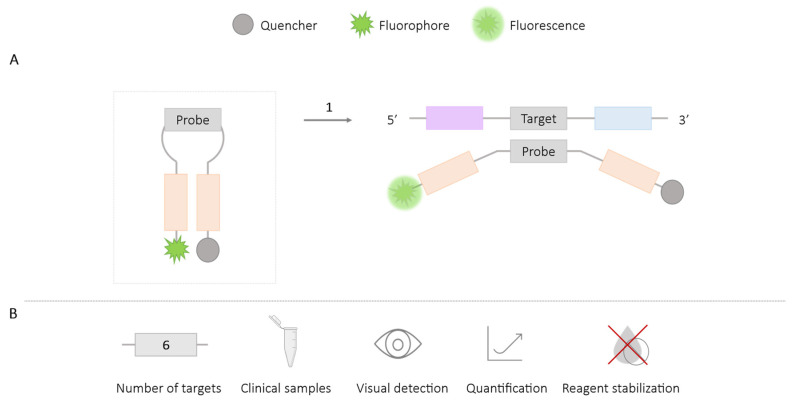
Molecular Beacon-LAMP Methodology. (**A**) Schematic representation of the molecular beacon methodology. In the box, a molecular beacon is depicted: a hairpin-like structure with a probe and two complementary sequences marked at the ends, one with a quencher and the other with a fluorophore. 1. LAMP reaction; fluorescence signal if there is amplification when the hairpin opens, separating the fluorophore and the quencher. (**B**) Application (maximum number of amplified targets and use of clinical samples), detection of amplification (visual at the end of the reaction or real-time quantitative), and adaptation to a ‘ready-to-use’ format (reagent stabilization).

The molecular beacon probe (25–45 bp), specific to the amplicons, is modified at one end with a fluorophore and at the other end with a quencher. Both ends are complementary and form a loop or hairpin structure that keeps the fluorophore and quencher close together, thereby suppressing the fluorescence signal. When the probe hybridizes with the target amplification product, it causes the opening of the hairpin, resulting in a fluorescence signal [57,58,59,60] (Figure 8).

The results of the assays can be monitored in real time using fluorescence reader devices [57]. The visual detection of results has also been achieved, either through subsequent analysis of band patterns in agarose gel electrophoresis that reveal specific bands associated with labeled probes [61], or by using microfluidic chips after the excitation of the fluorophores with UV light, resulting in a noticeable color change visible to the naked eye [59,60].

The application of molecular beacons has allowed for the detection of a single amplification target using plasmids for *Vibrio cholerae* [57] and *Vibrio parahemolyticus* [58], as well as the protozoan *Trypanosoma brucei* using genomic DNA [60]. Duplex LAMP format detection of HIV and HCV (Hepatitis C Virus) has also been achieved in clinical plasma samples [59], and even the amplification of six different targets for the detection of HIV, HCV, HBV (Hepatitis B Virus), HEV (Hepatitis E Virus), Dengue Virus, and West Nile Virus in clinical plasma samples [61].

#### 2.2.3. MD Probe 

**Figure 9:**
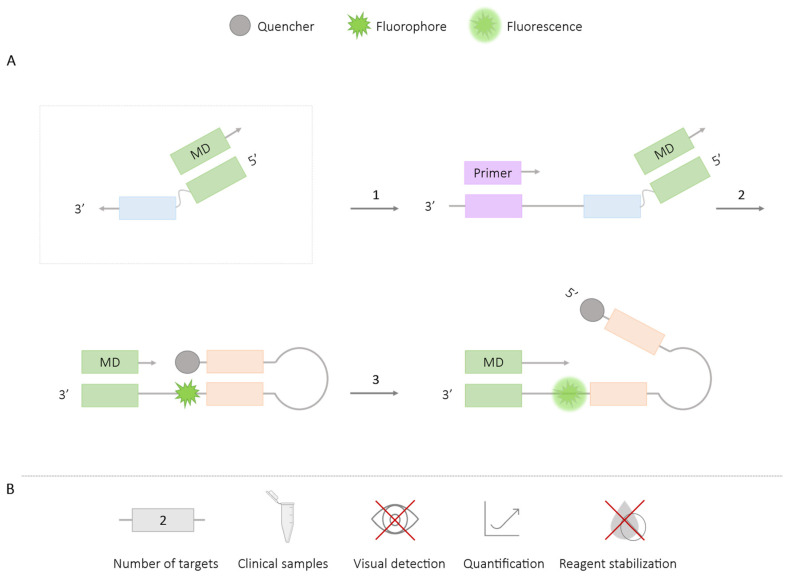
MD-LAMP Probe Methodology. (**A**) Schematic representation of the MD probe methodology. In the box, a bifunctional dimeric probe is depicted. 1. LAMP reaction and binding of the bifunctional dimeric probe. 2. Release of the mediator (MD); 3. Opening of the molecular beacon and fluorescence emission. (**B**) Application (maximum number of amplified targets and use of clinical samples), detection of amplification (visual at the end of the reaction or real-time quantitative), and adaptation to a ‘ready-to-use’ format (reagent stabilization).

The MD Probe or Mediator Displacement Probe methodology utilizes a bifunctional dimeric probe and a universal molecular beacon. The probe consists of a sequence partially complementary to a specific region of the target DNA, followed by a universal sequence at the 5′ end, and another sequence complementary to the universal sequence called the ‘universal mediator displacement’ (MD). During amplification, the MD is displaced, hybridizing with a specific sequence of the molecular beacon, causing it to open, and subsequently generating the fluorescence signal, which is detected by real-time fluorescence reader devices [22,62,63] (Figure 9).

The multiplex application of MD probes has been carried out for the simultaneous amplification of up to a maximum of two targets for the detection of HIV-1 and HTLV-1 (Human T-lymphotropic virus 1) [22], and for *Haemophilus ducreyi* and *Treponema pallidum* [22,62]. In the latter study for *H. ducreyi* and *T. pallidum*, the technique was validated using clinical samples collected from patients, including ulcer samples obtained with swabs [62].

### 2.3. Methods Using Restriction Enzymes or Endonucleases

**Figure 10:**
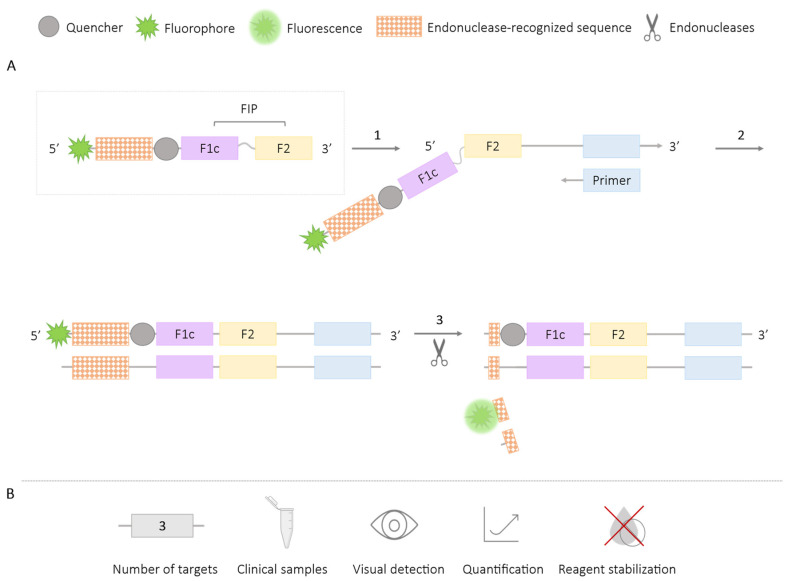
Methodology using Restriction Enzymes-LAMP. (**A**) Schematic representation of the methodology using restriction endonucleases. In the box, the first FIP primer is labeled with an endonuclease recognition sequence between a fluorophore and a quencher. 1. LAMP reaction. 2. Amplicons generated during amplification that include the endonuclease recognition sequence. 3. Digestion by endonucleases and emission of the fluorescence signal. (**B**) Application (maximum number of amplified targets and use of clinical samples), detection of amplification (visual at the end of the reaction or real-time quantitative), and adaptation to a ‘ready-to-use’ format (reagent stabilization).

An alternative approach for generating multiplex LAMP assays is by using restriction enzymes or endonucleases. The LAMP assay primers are designed by adding a recognition sequence for endonucleases or restriction enzymes, which will generate amplicons containing these sequences during amplification. Subsequently, the use of endonucleases will lead to the digestion of the amplified products. Following this methodology, different mechanisms for recognizing the digested products can be employed, enabling the simultaneous detection of multiple target sequences. One such mechanism is the Multiple Endonuclease Restriction Real-Time LAMP technology (MERT-LAMP), which involves labeling the endonuclease recognition sequence with a fluorophore and a quencher. During enzymatic digestion, these labels separate, generating a real-time fluorescence signal [64,65,66] (Figure 10). Another mechanism involves conducting agarose gel electrophoresis to visually distinguish the different specific amplicons. This is carried out after amplification and subsequent enzymatic digestion [67,68,69]. This latter mechanism is the only system that allows for the visual detection of the results in this methodology.

**Figure 11:**
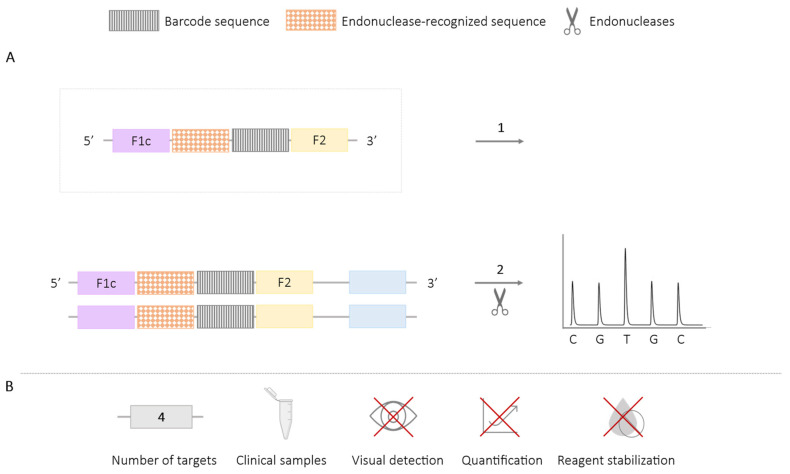
Methodology using Restriction Enzymes and Pyrosequencing-LAMP. (**A**) Schematic representation of the methodology using restriction endonucleases and pyrosequencing. In the box, the first FIP primer is labeled with an endonuclease recognition sequence and a “species-specific target barcode” sequence. 1. LAMP reaction and the generated amplicons include the endonuclease recognition sequence and the “species-specific target barcode” sequence. 2. Enzymatic digestion and pyrosequencing. (**B**) Application (maximum number of amplified targets and use of clinical samples), detection of amplification (visual at the end of the reaction or real-time quantitative), and adaptation to a ‘ready-to-use’ format (reagent stabilization).

The use of restriction endonucleases can be combined with pyrosequencing techniques for the specific detection of sequences. In this approach, primers labeled with endonuclease recognition sequences are also modified by adding “species-specific target barcodes” sequences, which are incorporated into the amplicons during amplification. After enzymatic digestion, the “barcode sequence” is decoded using pyrosequencing (Figure 11), a sequencing method that allows for the synthesis of short DNA sequences by detecting luminescence. This enables the detection of different targets [70]. Results using this technology do not allow for real-time reading or quantification of the amplification products.

The use of endonucleases for multiplex LAMP reactions has enabled the amplification of multiple targets, including the identification of three resistance genes to sulfonamides (*sul1*, *sul2*, *sul3*) in clinical samples isolated from *Enterobacteriaceae* [68]; the simultaneous detection of *Listeria monocytogenes* and *Listeria ivanovii*, validated in raw meat [65]; the detection of *Shigella* spp. and *Salmonella* spp. in milk samples [66]; the identification of *Vibrio parahaemolyticus* and *Vibrio vulnificus* in artificially contaminated oysters [64]; the detection of *Babesia bigemina* and *B. bovis* in bovine blood [67]; and the detection of *Chrysanthemum* B virus (CVB) and *Chrysanthemum* stunt viroid (CSVd) in chrysanthemum plant tissue [69]. Furthermore, the additional use of pyrosequencing methods has enabled the simultaneous detection of four targets for HIV, HCV, HBV, and *Treponema pallidum*, with validation of the analysis in clinical blood samples [70]. This demonstrates the capability of this approach to detect multiple pathogens or targets simultaneously in clinical samples, providing a valuable tool for diagnostic and research purposes.

### 2.4. Methods Using Nanoparticles

**Figure 12:**
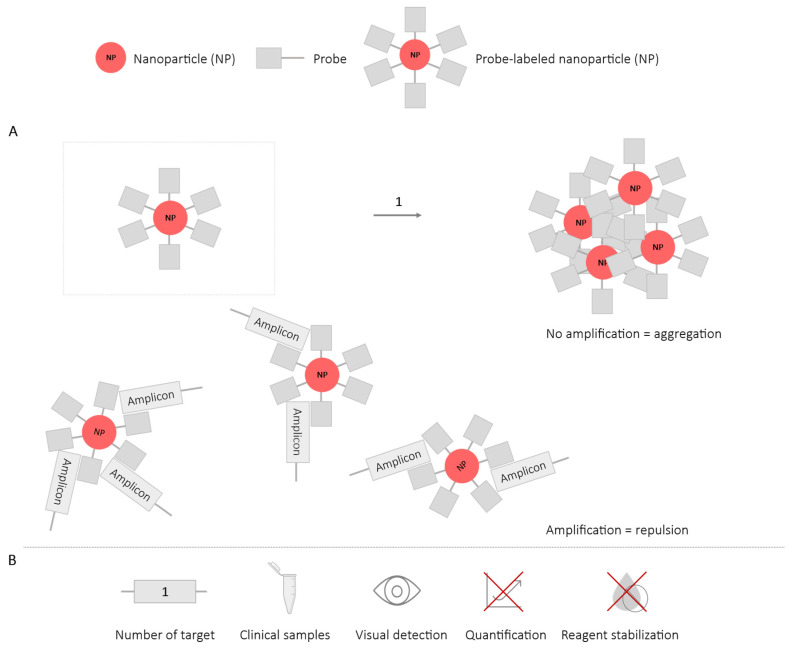
Nanoparticle-Based Methodology-LAMP. (**A**) Schematic representation of the functioning of the methodology employing nanoparticles. In the box, a nanoparticle (NP) is depicted, labeled with species-specific probes. 1. LAMP reaction; in the absence of amplification, NPs aggregate; in the presence of amplification, amplicons bind to the species-specific probes coating the NP, leading to NP repulsion. (**B**) Application (maximum number of amplified targets and use of clinical samples), detection of amplification (visual at the end of the reaction or real-time quantitative), and adaptation to a ‘ready-to-use’ format (reagent stabilization).

These methods employ nanoparticles (NPs) whose surfaces are modified with target-specific probes of the amplicons generated during amplification through LAMP. In the presence of amplification, the hybridization of the probes with the amplicons causes the repulsion of the NPs, keeping them evenly dispersed in the solution. If no amplification occurs (no probe–amplicon hybridization), the NPs tend to self-aggregate, resulting in a colorimetric signal (Figure 12). Various types of NPs exist, each exhibiting distinct color changes based on their state of aggregation or repulsion. Typically, gold nanoparticles (AuNPs) are the most commonly used, transitioning from red (in a state of repulsion) to blue/gray (in a state of aggregation), which is visible to the naked eye [71,72]. Other NPs, such as silver nanoparticles (AgNPs; yellow) or gold nanorods (AuNRs; cyan), are less frequently employed. The combined use of different types of nanoparticles (AgNPs, AuNPs, and AuNRs) for the detection of three targets through colorimetric differentiation has been experimentally explored, although it has not yet been employed in any amplification assay [73]. Nevertheless, this study suggests the potential combined use of NPs for multiplex LAMP applications, as the methodological procedure is conceptually similar to using a single type of NPs.

**Figure 13:**
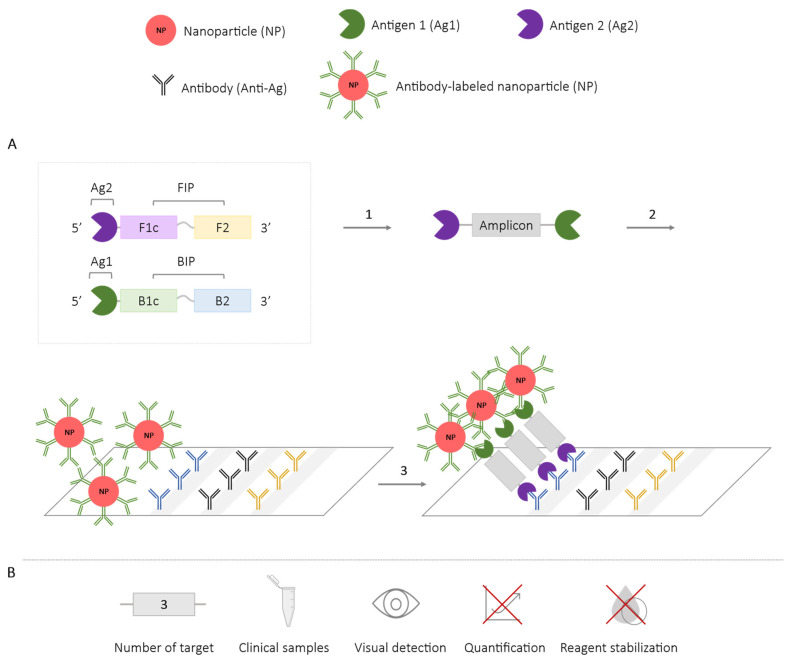
Nanoparticle-Based Methodology Combined with Immunochromatographic Strips-LAMP. (**A**) Schematic representation of the functioning of the methodology using nanoparticles with immunochromatographic strips. In the box, the first FIP primer is labeled with antigen 2 (Ag2), and the first BIP primer is labeled with antigen 1 (Ag1), both located at the 5′ end of the primer. 1. LAMP reaction and generation of amplicons labeled with antigens (Ag1 and Ag2). 2. Immunochromatographic strip with specific antibodies (anti-Ag2) and NPs labeled with anti-Ag1 antibody. 3. Binding of the amplicons generated in the LAMP reaction to the NPs (anti-Ag1) and on the immunochromatographic strip (anti-Ag2). (**B**) Application (maximum number of amplified targets and use of clinical samples), detection of amplification (visual at the end of the reaction or real-time quantitative), and adaptation to a ‘ready-to-use’ format (reagent stabilization).

To facilitate the multiplex application of this methodology, the combination of NPs with immunochromatographic strips or lateral flow assays has been employed. LAMP assay primers are labeled with two different antigens (Ag) that will be incorporated into the amplicons (Figure 13). Antigen 1 (Ag1), which is identical for all targets, will hybridize with anti-Ag1 antibodies coating the AuNPs. Antigen 2 (Ag2) is specific to each target and will have its corresponding antibodies on the immunochromatographic strip. As a result, the antigen-labeled amplicons will be retained on the immunochromatographic strips due to the binding of Ag2 and the antibody, while AuNPs bound to Ag1 will accumulate, showing a visible red band [74,75,76,77,78].

This methodology does not allow for the real-time detection or quantification of results since they are obtained exclusively at the end of the reaction. The maximum number of targets amplified using NPs has been three, for the simultaneous detection of *Pseudomonas aeruginosa* (ecfx) and two of its toxins (ExoS and ExoU) [74]. It has also been conducted in duplex LAMP format for the detection of *Enterococcus faecalis* and *Staphylococcus aureus*, validated in blood samples [75], the detection of two genes of SARS-CoV-2 validated in oropharyngeal samples [76], and the detection of two subtypes of Influenza A virus validated in nasopharyngeal samples [77]. Other studies have employed this methodology for the single detection of *Streptococcus iniae* in artificially infected zebrafish samples [71], Human Papillomavirus (HPV) in cervical tissue samples [72], and *Leptospira* spp. in bacterial culture samples [78].

### 2.5. Methods Using a Combination of Various Techniques

Combining LAMP technology with other diagnostic techniques can assist in the specific identification of sequences and, consequently, enable potential multiplex detection of different targets. Some of these methods include the combination with Enzyme-Linked Immunosorbent Assay (ELISA) and the combination with sequencing techniques.

#### 2.5.1. LAMP-ELISA 

In the LAMP-ELISA technique, LAMP primers are labeled with two molecules, digoxigenin and biotin, which are incorporated into the amplicons. After the LAMP reaction, the generated products are transferred to an ELISA plate previously coated with streptavidin (with a high affinity for biotin), and the labeled amplicons become immobilized on the surface of the plate. After performing the corresponding ELISA protocol, the results are detected in an automated plate reader, which provides absorbance values corresponding to the degree of hybridization of the amplicons with the specific probes fixed on the plate [79,80,81]. Performing the LAMP-ELISA requires extended processing times. The combination of both techniques has enabled the specific detection of sequences, although it has not been used for multiplex diagnosis to date. Some individual detection assays have been developed for a single target, such as the detection of *Mycobacterium tuberculosis* in patient sputum samples [79], or *Salmonella* spp. in artificially contaminated blood [81] and meat samples [80].

**Figure 14:**
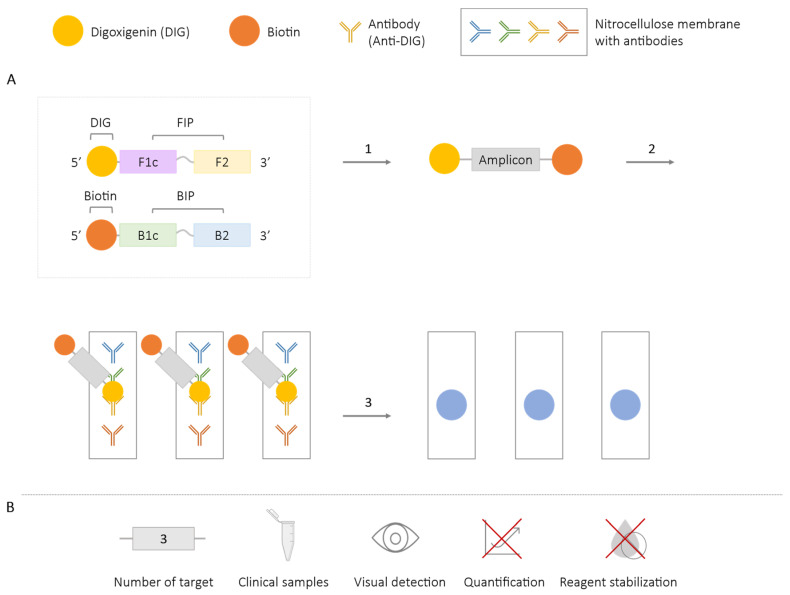
Combined LAMP-Dot-ELISA Methodology. (**A**) Schematic representation of the operation of the methodology that combines LAMP and Dot-ELISA. In the box, the first FIP is labeled with digoxigenin (DIG), and the first BIP is labeled with biotin, both labeled at the 5′ end. 1. LAMP reaction and generation of amplicons labeled with biotin and DIG. 2. Transfer of the LAMP product to the nitrocellulose membrane labeled with anti-DIG antibody. 3. Dot-ELISA. (**B**) Application (maximum number of amplified targets and use of clinical samples), detection of amplification (visual at the end of the reaction or real-time quantitative), and adaptation to a ‘ready-to-use’ format (reagent stabilization).

On the other hand, the combined use of LAMP and the Dot-ELISA alternative [82,83] has enabled the detection of *Taenia solium*, *T. saginata*, and *T. asiatica* in a single assay, validated in fecal samples [84]. The development is similar to the one described earlier, but different markers (digoxigenin or DIG, fluorescein isothiocyanate or FITC, and tetramethylrhodamine or TAMRA) are used, which are integrated into different species-specific amplicons. Instead of a coated plate, a nitrocellulose membrane is used, coated with specific antibodies for DIG, FITC, and TAMRA. A positive result is observed as a visible colored spot on the membrane [84] (Figure 14). In neither of the two combined assays, neither LAMP-ELISA nor LAMP-Dot-ELISA, is a signal obtained for real-time detection and quantification of the results.

#### 2.5.2. LAMP-Sequencing

**Figure 15:**
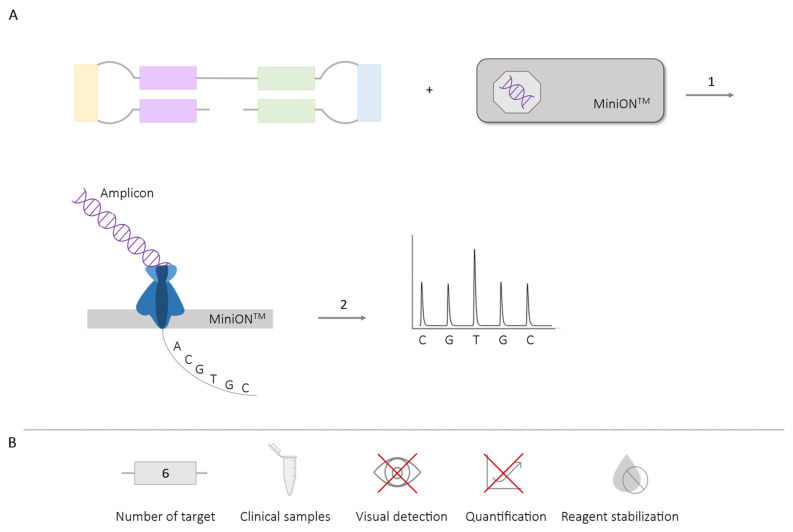
Combined LAMP-Sequencing Methodology. (**A**) Schematic representation of the operation of the methodology that combines LAMP with sequencing using MinION^TM^. In the box, LAMP and a MinION^TM^ sequencer are depicted. 1. LAMP reaction; the generated amplicons are transferred to the MinION^TM^ sequencer. 2. Representation of the results obtained from sequencing. (**B**) Application (maximum number of amplified targets and use of clinical samples), detection of amplification (visual at the end of the reaction or quantitative in real-time), and adaptation to a “ready-to-use” format (reagent stabilization).

Sequencing the amplicons generated in LAMP assays allows for detailed sequence analysis, aiding in the identification of multiple targets. The previously mentioned pyrosequencing technique (see Section 2.3) requires the labeling of specific amplicons for subsequent identification. However, using nanopore sequencing with a portable MinION^TM^ sequencer (Oxford Nanopore Technologies, Oxford, UK) does not require any labeling or the use of additional probes for identification (Figure 15). Nevertheless, this process requires extended periods of time as it necessitates prior DNA library preparation, purification [85,86] and the sequencing protocols can last from 24 to 48 h [85,86,87,88,89].

The use of MinION^TM^ devices has allowed for multiplex application in the simultaneous amplification of six *Plasmodium* species in blood samples from patients [89]; four different serotypes of Dengue virus in blood samples from patients [85]; artemisinin resistance mutation in *Plasmodium falciparum* in blood samples from patients [88], and different genotypes of Chikungunya virus in serum samples from patients [86]. Moreover, the latter study was conducted using LAMP reagents that had been previously stabilized through a drying protocol and stored at room temperature for two months [86].

### 2.6. Other Methods

There are other simpler alternatives that can be applied to multiplex LAMP diagnostics, such as melting curve analysis or agarose gel electrophoresis. The differentiation of the amplified products by melting curve analysis allows for the distinction of different products based on their melting temperatures. The results depend on the primer’s own melting temperatures, which vary depending on their GC content and must be different enough to be distinguished in the melting curve obtained at the end of the reaction. Therefore, this type of analysis cannot always be carried out [90,91]. This strategy has allowed for the application of LAMP in a duplex format to detect of *Salmonella* spp. and *Vibrio parahaemolyticus* in artificially contaminated milk samples [90], and *Leishmania donovani* and *Mycobacterium leprae* in patient tissue biopsies [91].

The differentiation of amplified products for multiplex LAMP detection using agarose gel electrophoresis can follow two strategies. On the one hand, the differentiation of the banding patterns of each specific LAMP by different ladder banding. This method has been used for the simultaneous detection of *Salmonella* spp. and *Shigella* spp. in artificially contaminated milk samples [92]. On the other hand, by labeling one of the internal primers in each primer set with a different fluorophore that allows for specific banding differentiation by colorimetry. This strategy has been applied for the simultaneous detection of *Plasmodium berghei* and *Dirofilaria immitis* in infected mosquitoes [93].

Other more complex methodologies involve the development of microfluidic chips integrated with LAMP assays, which have the potential to provide rapid diagnostics by combining genetic material extraction, reaction processing, and real-time reading and/or result interpretation [94,95,96,97]. These types of assays have been used for multiplex LAMP detection of up to five species, including *Streptococcus agalactiae*, *Enterococcus faecalis*, *Gardnerella vaginalis*, *Candida albicans*, and *Chlamydia trachomatis* in lower genital tract samples [97], as well as specific genes from camels, cows, mares, yaks, and goats for species identification from milk samples [95]. They have also been used for the simultaneous detection of *Streptococcus pneumoniae* and *Mycoplasma pneumoniae* in oropharyngeal and bronchoalveolar samples from patients [94], and of bacteriophage λ and *Escherichia coli* using genomic DNA [96].

Table 1 provides a summary of the various sequence-dependent methodologies, used in multiplexing LAMP described here, that have proven useful in the simultaneous detection of multiple targets, comparing the number of targets detected, species/targets amplified, application in clinical samples, visual detection, as well as quantification and reagent stabilization.

Regarding the number of amplified targets, most methodologies have demonstrated their application to amplify two or more targets, with molecular beacon (Section 2.2.2) and LAMP-sequencing (Section 2.5.2) standing out, as they have demonstrated the ability to amplify a maximum of six targets per reaction [61,89]. Exceptions are the guanine quenching (Section 2.1.4) and NPs (Section 2.4) methodologies, which have only been able to amplify a single target. However, these two methods could potentially be applied to a multiplex format by using the combination of primers labeled with different fluorophores (guanine quenching) and by using different nanoparticles that can be colorimetrically differentiated (NPs) [73]. In practice, using multiplexing methods, the potential increase to a higher number of amplified targets would be constrained by the methodology itself. For instance, with the QUASR method (Section 2.1.2), increasing the number of targets to detect would result in overlapping colors, making it challenging to differentiate the colorimetric results. In the melting curve method (Section 2.6), distinguishing multiple targets is influenced by the primer temperatures, which need to be sufficiently different to be distinguished at the end of the reaction [90,91]. This would complicate the differentiation of more than two targets, as the primer temperatures used in the amplification are typically very similar. The differentiation of more than two targets amplified for multiplex LAMP using the agarose gel electrophoresis (Section 2.6) is conditioned by a distinct band ladder pattern generated in the amplification assay, which can be challenging as they may appear quite similar. Nevertheless, the fluorophore labeling of one of the primers used in LAMP, following the methodology of Anouma et al. 2010 [93], could potentially be applied for the amplification of a larger number of targets for possible differentiation. The MD-LAMP probe methodology (Section 2.2.3), which has been applied for the simultaneous amplification of up to a maximum of two targets, theoretically could be applied to a greater number of targets since the results are detected using conventional real-time fluorescence readout devices. The number of targets to be detected would be limited by the number of excitation and emission channels in the real-time fluorescence readout device capable of detecting the signals generated by the different fluorophores. The potential use of this methodology in POC for the detection of multiple targets would be more limited, as portable fluorescence readout devices generally have only two channels for reading emission and excitation for fluorophores and are therefore limited to distinguishing only two targets in a single multiplex LAMP reaction.

With the exception of the methods using OSD probes (Section 2.2.1) and NPs (Section 2.4), all other multiplexing LAMP methodologies have been applied in the analysis of clinical samples, although work is still scarce and poorly reproduced. The target sequences for amplification are diverse, with a predominance of publications focusing on viruses and bacteria. Protozoa studies are mainly centered around *Plasmodium* spp. [29,51,88,93]. When it comes to Neglected Tropical Diseases (NTDs), virus-related research is more prevalent than other diseases, particularly Chikungunya and Dengue [30,31,33,43,61,85,86]. A study conducted in 2021 by Joshi and colleagues achieved the simultaneous detection of *Leishmania donovani* and *Mycobacterium leprae*, the causative agents of leishmaniasis and leprosy, respectively [91]. Helminth studies are still scarce, despite the variety of species causing NTDs and the high rate of coinfections by various species in endemic areas. Recently, a duplex DARQ-LAMP assay was developed for the simultaneous detection of *Schistosoma mansoni* and *Strongyloides* spp. [28]. A single study managed to simultaneously amplify three *Taenia* species: *Taenia solium*, *T. saginata*, and *T. asiatica* [84]. The development and application of these multiplexing methodologies in the analysis of clinical samples would aid in the validation of the different techniques and promote reproducibility by other laboratories and research centers.

As is well known, the visualization of the results with the naked eye by colorimetric change is one of the main advantages of nucleic acid amplification by LAMP [9]. Several of the multiplexing LAMP methodologies presented here also facilitate such visualization, either immediately at the end of the reaction or, in most cases, by applying post-reaction procedures necessary to make the results visible. Thus, only the NPs methodology (Section 2.4) could facilitate the direct visualization of the results at the end of the reaction [71,72] while the QUASR (Section 2.1.2) and assimilation probes (Section 2.1.5) methodologies require illumination of the reaction tubes with led or UV light to visualize the colorimetric results [30,31,43,45,46]. Additionally, for the QUASR methodology, the possibility of enhancing or brightening the colorimetric results by using apps designed for use with a smartphone has also been described [31,32].

The FLOS (Section 2.1.3) [35,36] and assimilation probes (Section 2.1.5) [47] methodologies have utilized the post-reaction addition of the polymer polyethyleneimine (PEI), which precipitates the amplification products after brief centrifugation, generating a colored pellet visible to the naked eye under UV light. On the other hand, simple methods such as agarose electrophoresis can be used to visualize amplification results obtained with molecular beacon methodology (Section 2.2.2) [61] or with LAMP products subsequently treated with enzymatic digestion as in the case of methods using endonucleases (Section 2.3) [67,68,69].

In addition, as mentioned above, by combining multiplex LAMP technology with other diagnostic techniques, the visual detection of different targets is also possible. In this way, the results obtained with the molecular beacon methodology (Section 2.2.2) can be visualized using microfluidic chips (Section 2.6) [59,60]. The combination of NPs with immunochromatographic strips (Section 2.4) allows the results to be observed thanks to the visible banding generated on the strips [74,75,76,77,78]. The combined LAMP-Dot-ELISA methodology (Section 2.5.1) allows for the visualization of the results on a nitrocellulose membrane coated with specific antibodies [84]. The need for post-reaction processing for the visualization and interpretation of the results after a multiplex LAMP reaction not only increases reaction time, but also technical skills, infrastructure and costs by requiring additional procedures or mechanisms beyond those needed for multiplex LAMP reactions.

The quantification of multiplexed LAMP amplification results can be performed in those methodologies that use typically modified primers (Section 2.1) with fluorophores and those using universal probes (Section 2.2) to allow for real-time measures of the fluorescence signal. The exception is the QUASR methodology (Section 2.1.2) which, due to the mechanism of the technique itself, is limited to a final signal after amplification, which does not allow for real-time quantification. When methods using restriction enzymes (Section 2.3), the recognition sequence of the endonucleases can be labelled with a fluorophore that generates a quantifiable fluorescence signal during the enzymatic digestion process [64,65,66]. Real-time quantification is undoubtedly a great advantage, but it still requires the use of expensive fluorescence readout devices (portable or benchtop) or the development of microfluidic chips (Section 2.6).

The stabilization of reaction components at ambient temperature represents a significant advancement in eliminating the need for the cold chain and bringing LAMP diagnostics closer to the POC. However, this feature has not been widely implemented in various multiplexing LAMP methodologies. The stabilization by drying LAMP reaction components in the presence of trehalose and subsequent storage until use has been utilized in the LAMP-sequencing (Section 2.5.2) method [86] and more recently by our group in the DARQ-LAMP methodology (Section 2.1.1) [28]. In both cases, the desiccated reagents remained viable after two months of storage at room temperature. Nevertheless, using desiccated DARQ-LAMP mixtures, an increase in amplification times and a reduction in fluorescence signals have been observed compared to the fresh liquid DARQ-LAMP mixtures in the simultaneous amplification of DNA from *Schistosoma mansoni* and *Strongyloides* spp. [28]. This effect has also been observed when working with dry-LAMP mixtures for single target amplification [16,98]. On the other hand, reagents stabilized by lyophilization have been used in methodologies employing assimilation probes (Section 2.1.5) [43,45,46,49,50] and OSD probes (Section 2.2.1) [52], achieving functionality of the reaction up to one month and 100 days after storage, respectively. The stability at ambient temperature of lyophilized multiplex LAMP mixtures appears to be better than that of desiccated mixtures based on the use of trehalose as a cryoprotectant to produce ready-to-use functional reaction mixtures. However, the lyophilization process is much more laborious and requires sophisticated equipment and higher additional costs than the drying process. The optimization and improvement of the long-term stability of multiplex LAMP at ambient temperatures, for real application as a POC in field conditions, is still needed.

Finally, considering the characteristics examined and compared, we cannot definitively determine which LAMP multiplexing methodology is the best overall. Each methodology has its advantages and drawbacks, and the choice between them will depend on specific interests. It is true that the methodology using assimilating probes (Section 2.1.5) meets all the characteristics mentioned here. However, other methodologies, such as DARQ (Section 2.1.1), FLOS (Section 2.1.3), molecular beacon (Section 2.2.2), endonucleases (Section 2.3), and microfluidic chip use (Section 2.6) are very close to meeting all the criteria. It is highly likely that in the near future, advancements in these technologies or in complementary procedures will improve their performance and applicability.

## 3. Conclusions and Future Perspectives

The POC applicability of LAMP technology is one of its most notable qualities and is gaining increasing attention among researchers. However, simple and portable instruments are required. The fluorescence-based multiplexed detection of LAMP methods designed for multiple target detection is time-consuming for the primer optimization and probe design needed to fine-tune the assay. In addition, since fluorescence detection is required, adapted systems or ad hoc development are necessary for use as POC diagnostic tests. To address this potential limitation, some studies have utilized microfluidic chips [94,95,96,97] or portable LAMP boxes designed for the easy interpretation of results using photographic filters [30,31,32,43,45]. Smartphone apps have also been developed to aid in the interpretation of results [31,32]. Other commercial devices, such as those offered by OptiGene (UK), come equipped with batteries for autonomous use without the need for electricity, and are capable of real-time fluorescence detection [42,44]. On the other hand, the use of lateral flow strips facilitates the application of multiplex LAMP methodologies, as it simplifies their execution and the interpretation of results [74,75,76]. However, despite the variety of methodologies and studies performed, no multiplex LAMP method has yet been applied as a POC diagnostic test under field conditions.

In summary, there are different multiplexing LAMP methodologies for the simultaneous detection of different targets that are distinguished by their different application characteristics. This review aims to provide an overview of these methodologies that can serve as a guide for the selection of the most appropriate methods. Overall, the current merits of multiplex LAMP technology outweigh its disadvantages. We must continue to work on its development.

## Figures and Tables

**Table 1:** Summary of the sequence-dependent methodologies used in LAMP multiplexing indicating the number of simultaneously detected targets, amplified species/targets, application in clinical samples, visual detection, quantification and reagent stabilization.

Methodologies	Number of Targets	Species/Target Amplified	Application in Clinical Samples	Visual Detection	Quantification	Reagent Stabilization
DARQ	4	*Escherichia coli*, *Caenorhabditis elegans*, bacteriophage λ, hBRCA1 [24] Methicillin-resistant *Staphyloccus aureus* genes (femB, mecA, spa) [26] *Salmonella* spp. [27] *Plasmodium* spp., *P. vivax*, *P. falciparum*, Human actin [29] *Schistosoma mansoni*, *Strongyloides* spp. [28]	✓	✗	✓	✓
QUASR	2	West Nile virus, Chikungunya [30] Zika, Chikungunya [31] Yellow fever virus, Dengue [33]	✓	✓	✗	✗
FLOS	3	Varicella-zoster virus [34] *Trialeurodes vaporariorum*, *Bemisia tabaco* (MEAM1) and *B. tabaci* (MED) [35] *Fulviformes umbrinellus* and *Fomitiporia torreyae* [36]	✓	✓	✓	✗
Guanine *quenching* *	1	IV [37] RSV [37] *Nitrosomonas europaea* [39] MERS-CoV [40]	✓	✗	✓	✗
Assimilation probes or primer fluorescence probes	3	Zika, Dengue, Chikungunya [43,45] SARS CoV-2 and hARNasa P [46] *Salmonella enterica* and *Enterobacteria phage λ* [42] *Ralstonia salanacearum* and *R. solanacearum* R3B2 [41,42] Mitochondrial cytochrome b gene (cow and goat) [44] HIV-1 [48,49,50] Sex embryos [47]	✓	✓	✓	✓
OSD probes	4	HSV1 and *P. falciparum* [51] Polymorphic detection in *BRAF* gen [51] Human fecal contamination *(Bacteroides HF183)* [52] *Wolbachia* spp. [54] MERS-CoV [55] Zika (4 genotypes) [56]	✗	✗	✓	✓
Molecular beacon	6	*Vibrio cholerae* [57] HIV, HBV, HCV, HEV, Dengue and West Nile virus [61] HIV and HCV [59] *Vibrio parahaemolyticus* [58]	✓	✓	✓	✗
MD probe	2	HIV-1 and HTLV-1 [22] *Haemophilus ducreyi* and *Treponema pallidum* [22] *T. pallidum* and *H. ducreyi* [62]	✓	✗	✓	✗
Endonucleases	3	Sulfonamide resistance genes (sul1, sul2, sul3) [68] *Babesia bigemina* and *B. bovis* [67] *Crysanthemum virus B* (CVB) and *Crysanthemum stunt viroid* (CSVd) [69] *Listeria monocytogenes* and *L. invanovii* [65] *V. parahaemolyticus* and *Vibrio vulnificus* [64] *Shigella* spp. and *Salmonella* spp. [66]	✓	✓	✓	✗
Endonucleases + pyrosequencing	4	HBV, HCV, HIV and *T. pallidum* [70]	✓	✗	✗	✗
Nanoparticles *	1	HPV [72]	✗	✓	✗	✗
Nanoparticles + lateral flow assay	3	*Streptococcus iniae* [71] *Pseudomonas aeruginosa* (ecfx) and toxin ExoS and ExoU [74] Influenza A virus (2 subtypes) [77] *Leptospira* spp. [78] *Enterococcus fecalis* and *S. aureus* [75] SARS-CoV-2 (2 genes) [76]	✓	✓	✗	✗
LAMP-ELISA/dot-ELISA	3	*Salmonella* spp. [80,81] *Mycobacterium tuberculosis* [79] *Taenia solium*, *T. saginata* and *T. asiatica* [84]	✓	✓	✗	✗
LAMP- sequencing	6	Dengue (4 serotypes) [85] *P. falciparum* (artemisinin resistance mutation) [88] *Plasmodium* (6 spp.) [89] Chikungunya (genotypes) [86]	✓	✗	✗	✓
Melting curve	2	*Salmonella* spp. and *V. parahaemolyticus* [90] *Leishmania donovani* and *Mycobacterium leprae* [91]	✓	✗	✓	✗
Gel electrophoresis	2	*Salmonella* spp. and *Shigella* spp. [92] *Plasmodium berghei* and *Dirofilaria immitis* [93]	✓	✓	✗	✗
Microfluidic chips	5	*Streptococcus pneumoniae* and *Mycoplasma pneumoniae* [94] Specific genes cow, camel, goat, horse and yak [95] *E. coli* and bacteriophage *λ* [96] *Streptococcus agalactiae*, *Enterococcus faecalis*, *Gardnerella vaginalis*, *Candida albicans* and *Chlamydia trachomatis* [97]	✓	✓	✓	✗

* methods that have not been applied in multiplex format. ✓: Indicates that method has been tested on clinical samples, results are visually detectable, allows for the quantification of results and has been tested with stabilized reagents. ✗: Indicates not tested on clinical samples, no visual detection of results, does not allow for quantification and has not been tested with stabilized reagents. BRCA1, human breast cancer gene; IV, influenza virus; RSV, respiratory syncytial virus; HSV1, herpes simplex virus 1; HIV, human immunodeficiency virus; HBV, Hepatitis B virus; HCV, Hepatitis C virus; HEV, Hepatitis E virus; HTLV-1, human T-lymphotropic virus 1; HPV, human papillomavirus.

## Data Availability

All data generated or analyzed during this study are included in the article.

## Abbreviations

Ag	Antigen
BRAF oncogene	B-Raf proto-oncogene serine/threonine-protein kinase
CRISPR-Cas	Clustered Regularly Interspaced Short Palindromic Repeats and associated protein Cas
CVB	*Chrysanthemum* B virus
DARQ	Detection of Amplification by Release of Quenching
DIG	Digoxigenin
ELISA	Enzyme-Linked Immunosorbent Assay
FITC	Fluorescein isothiocyanate
FLOS	Fluorescence of Loop primer upon Self-dequenching
hBRCA1	gene *human* breast cancer 1 gene
HBV	Hepatitis B Virus
HCV	Hepatitis C Virus
HEV	Hepatitis E Virus
HIV	Human immunodeficiency virus
HPV	Human Papillomavirus
HTLV-1	Human T-lymphotropic virus 1
iNAATs	Isothermal nucleic acid amplification techniques
IV	Influenza virus
LAMP	loop-mediated isothermal amplification
LED light	Light-emitting diode light
LFA	lateral flow assays
MD	Mediaton displacement
MERS-CoV	Middle East Respiratory Syndrome Coronavirus
MERT-LAMP	Multiple Endonuclease Restriction Real-Time LAMP technology
mLAMP	multiplex LAMP
NPs	Nanoparticles
NTDs	Neglected Tropical Diseases
PEI polymer	Polyethylenimine or polyaziridine polymer
POC	point-of-care
qPCR	Quantitative polymerase chain reaction
QPD	Quencher Probe Duplex
QUASR	Quenching of Unincorporated Amplification Signal Reporters
RSV	Respiratory Syncytial Virus
SARS-CoV-2	Severe Acute Respiratory Syndrome Coronavirus 2
TAMRA	Tetramethylrhodamine
UV light	Ultraviolet light
